# Postnatal ontogenesis of clock genes in mouse suprachiasmatic nucleus and heart

**DOI:** 10.1186/1476-511X-9-22

**Published:** 2010-03-05

**Authors:** Jie Huang, Chao Lu, Sifen chen, Luchun Hua, Ruizhe Qian

**Affiliations:** 1 Department of Physiology and Pathophysiology, Shanghai Medical College Fudan University, Shanghai 200032, PR China; 2 Department of Surgery, Huashan Hospital affiliated to Fudan University, Shanghai 200040, PR China

## Abstract

**Background:**

The master clock within the hypothalamic suprachiasmatic nucleus (SCN) synchronizing clocks in peripheral tissues is entrained by the environmental condition, such as the light-dark (LD) cycle. The mechanisms of circadian clockwork are similar in both SCN and peripheral tissues. The aim of the present work was to observe the profiles of clock genes expression in mouse central and peripheral tissues within postnatal day 5 (P5). The daily expression of four clock genes mRNA (Bmal1, Per2, Cry1 and Rev-erb alpha) in mouse SCN and heart was measured at P1, P3 and P5 by real-time PCR.

**Results:**

All the studied mice clock genes began to express in a circadian rhythms manner in heart and SCN at P3 and P5 respectively. Interestingly, the daily rhythmic phase of some clock genes shifted during the postnatal days. Moreover, the expressions of clock genes in heart were not synchronized with those in SCN until at P5.

**Conclusion:**

The data showed the gradual development of clock genes in SCN and a peripheral tissue, and suggested that development of clock genes differed between in the SCN and the heart. Judging from the mRNA expression, it was possible that the central clock synchronized the peripheral clock as early as P5.

## Introduction

In mammals, the circadian pacemaker is in the hypothalamic suprachiasmatic nucleus (SCN), which regulates most physiological and behavioral circadian rhythms with a period of approximately 24 hours [1,2]. The master circadian system is mainly entrained to the 24-h day by light-dark (LD) cycle [3]. Apart from SCN, it is shown that peripheral tissues (such as heart, liver, fat, muscle, etc) have independent circadian oscillators [4,5]. The SCN synchronizes the peripheral clocks via incompletely understood nervous and humoral signals so that they achieve optimal phase relationships in daily environmental cycle [6-8]. The molecular mechanism of circadian oscillations is considered to be composed of the interlocking autoregulatory transcriptional and translational feedback loops consisting of a set of core clock genes which is Clock gene, Bmal1 gene, three Period genes(Per1, Per2, Per3), two Cryptochrome genes(cry1, cry2), CKIε and Rev-Erbα [9,10]. Briefly, two basic helix-loop helix (bHLH)-PAS proteins CLOCK and BMAL1 forming heterodimers positively activate transcription of Per, Cry and Rev-Erbα genes and drive their rhythmic expression. In the cytoplasm, PER and CRY proteins form complexes translocate back to the nucleus where they inhibit CLOCK/BMAL1 activates. In addition, the phosphorylation of PER proteins by CKIε regulating their stability also plays important role in the circadian expression [1,11-15]. Rev-Erbα could regulate the expression of Bmal1 by encoding an orphan nuclear receptor to periodically represses Bmal1 transcription and retinoic acid receptor-related orphan receptor-α competes with Rev-Erbα to activate Bmal1 transcription[16,17]. The SCN and the peripheral tissues share the same predominantly molecular mechanism of circadian rhythm [18,19].

Recent studies have suggested that the gradual development of clock genes rhythmically express in the SCN and peripheral tissues of mammals during ontogenesis. There are no rhythms of clock genes at the embryonic day (E) 19 but the postnatal day (P) 3 in the rat SCN [3]

In the rat heart, circadian expression of Per1, Bmal1 and Dbp mRNAs started between P2 and P5 [20]. While in the mouse, through the SCN matures gradually during postnatal life and the melatonin-dependent peripheral pars tuberalis oscillator matures during foetal life[21], there was no report which showed the exact day when the clock genes started to show rhythmicity in mouse. Moreover, little is known about development of clock genes expression in the mouse SCN and peripheral tissue between P1 and P5 in detail. The aim of the present work is to ascertain the exact day when the clock genes expressed with circadian rhythms in the mouse SCN and heart by partly filling the gap of our knowledge that daily profiles of clock genes expression within P5. Therefore, we observed developmental rhythms of clock genes which are Per2, Bmal1, Cry1 and Rev-erbα in the mouse SCN and heart at P1, P3, P5.

## Results

### Daily expressions of Clock genes mRNA

**Figure 1:**
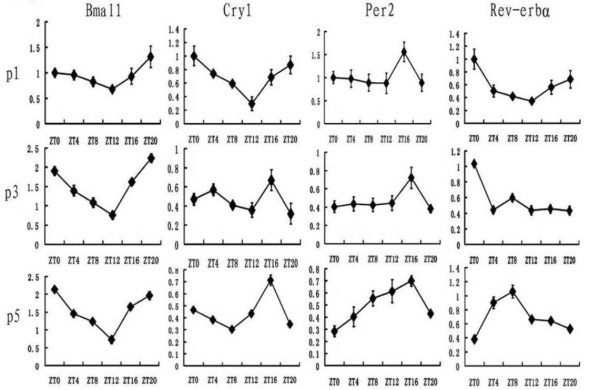
**Daily profiles of expression of clock genes Bmal1, Cry1, Per2 and Rev-erbα in the mouse SCN at the postnatal day 1 (P1), P3, P5**. The mRNA level was normalized to GAPDH mRNA. The value at ZT0 for P1 mice was defined as 1. Values represent the mean ± SEM (n = 3 per time point).

Fig. 1 show daily profiles of Bmal1, Cry1, Per2 and Rer-erbα expression in the SCN of 1-, 3- and 5-day-old mice.

At P1, the one-way ANOVA revealed some variation on expression of Cry1 (P < 0.001), Rev-erbα (P < 0.01) and Per2 (P < 0.05), but not on expression of Bmal1. Cry1 mRNA expression at ZT12 was significantly lower than those at ZT0, ZT4, ZT20 (P < 0.001) and ZT16 (P < 0.05), but not that at ZT8. For Rev-erbα mRNA expression, the level at ZT0 was significantly higher than ZT4, ZT8, ZT12 (P < 0.01) and ZT16 (P < 0.05). Moreover, the level at ZT20 was higher than that at ZT12 (P < 0.05). For Per2, there was no clear circadian rhythm, although the one-way ANOVA revealed some variation throughout the day, it's level at ZT16 was higher than those at ZT4, ZT8, ZT12 and ZT20 (P < 0.05).

At P3, a significant time effect was present for Bmal1, Cry1 and Rev-erbα. Bmal1 mRNA level began to decline between ZT20 and ZT0, and reached the minimal level betweenZT8 and ZT12. Bmal1 expression at ZT 12 was significant lower than values at any other time except at ZT8 (P < 0.01). Cry1 mRNA peaked at ZT16 (P < 0.05, between ZT8 and ZT16). Rev-erbα expression at ZT0 was significantly higher than any other time point (P < 0.001), however, the level at ZT8 was higher than the other time points expect ZT0 (P < 0.05).

At P5, all the studied clock genes expressed in the circadian manner, as the one-way ANOVA revealed significant rhythms in their mRNA expression. Bmal1 level started to decline around ZT0, and reached the minimal level at ZT12 (P < 0.001, between ZT0 and other time points), than rise fast between ZT12 and ZT16 (P < 0.001). Cry1 mRNA peaked at ZT16 (vs. ZT4, ZT8 and ZT20, P < 0.001). Per2 expression at ZT16 was significantly higher than ZT0, ZT4 and ZT20 (P < 0.01), but no differences among ZT8 and ZT16. The peak of Rev-erbα mRNA level around ZT8 was significantly higher than others (P < 0.01) except ZT4. A significant increase above ZT0 value occurred at ZT4 (P < 0.001) and a significant decline from high daytime value occurred at ZT12 (p = 0.001). Similar to adult animals, the Bmal1 expression was roughly in an opposite phase to the daily oscillator of Per2 mRNA.

**Figure 2:**
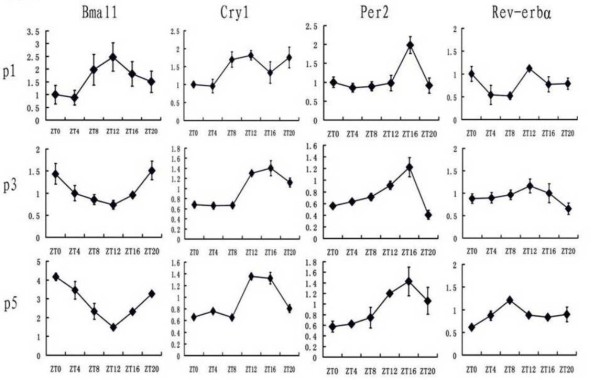
**Daily profiles of expression of clock genes Bmal1, Cry1, Per2 and Rev-erbα in the mouse heart at the postnatal day 1 (P1), P3, P5**. The mRNA level was normalized to GAPDH mRNA. The value at ZT0 for P1 mice was defined as 1. Values represent the mean ± SEM (n = 3 per time point).

Fig. 2 show daily profiles of Bmal1, Cry1, Per2 and Rer-erbα expression in the hearts of 1-, 3- and 5-day-old mice.

At P1, the one-way ANOVA revealed significant time effect on expression of the studied clock genes except Bmal1 (P < 0.05), but no clear circadian rhythm throughout the day. Cry1 level started to rise after ZT8, and significant increase above the ZT8 value occurred between ZT12 and ZT20 (P < 0.05). Rev-erbα expression at ZT4-8 was significantly lower than levels at other ZTs (P < 0.05). For Per2 mRNA, one-way ANOVA revealed that the peak in Per2 expression at ZT16 was significantly higher than values at any other time (P < 0.05).

At P3, a significant time effect was present for Bmal1, Cry1 and Per2 mRNA (P < 0.01) and Rev-erbα (P < 0.05). Moreover, the four clock genes started to express roughly daily rhythms. Bmal1 level began to decline after ZT0 and reached trough value at ZT12 (P < 0.01, between ZT0 and ZT12). Cry1 expression at ZT12 was significantly higher than ZT0-8 (P < 0.05) then declined after ZT16. Per2 mRNA at ZT16 was significantly higher than ZT0, ZT4, ZT8 and ZT20 (P < 0.001) and ZT12 (P < 0.05). Rev-erbα mRNA expression at ZT12 was significant higher than ZT20 (P < 0.05).

At P5, there were significant circadian rhythms of all the studied clock genes mRNA. The one-way ANOVA revealed significant time effect on the four clock genes expression. Bmal1 mRNA at ZT0, ZT4 and ZT20 was significantly higher than ZT12 (P < 0.01) and ZT0 level was significantly higher than ZT20 (P < 0.05). Thus, the Bmal1 level started to rise between ZT12 and ZT16, reached the maximal level at ZT0, and declined around ZT0. Cry1 level peaked between ZT12-16 (vs. ZT0-8 and ZT20, respectively; P < 0.001) and began to decline around ZT16. Per2 mRNA expression increased slowly between ZT and ZT8 then rise faster after ZT8, reached the maximal level between ZT12 and ZT16, and declined after ZT16. Per2 level at ZT12 and ZT16 was significantly higher than ZT0-8(P < 0.05). Rev-erbα expression peaked at ZT8 (P < 0.001, between ZT8 and ZT0). Rev-erbα mRNA began to rise after ZT0 and ZT4 was higher than ZT0 (P < 0.05). Moreover, the level declined between ZT8 and ZT12 (P < 0.05, between ZT8 and ZT12). The Bmal1 expression was roughly in an opposite phase to the daily oscillator of Per2 mRNA.

### Synchronization of circadian rhythms between the SCN and heart

According to the Fig. 1 and Fig. 2, we could find that phase of some clock genes shifted during the postnatal development in the mouse SCN and heart. In the SCN, at P1, the Cry1 mRNA peaked at ZT0. Then it peaked at ZT16 at P3 and P5. For Rev-erbα mRNA, the peak shift from ZT0 at P1 and P3 to ZT8 at P5. In the heart, for Bmal1, the timepoint of ZT12 which was the peak at P1 while developed to the trough at P3 and P5. For Cry1, the mRNA expression level had no exact peak at P1, but at P3 and P5, it peak between ZT12 and ZT16. For Rev-erbα, the peak shift from ZT12 at P3 to ZT8 at P5. Our data showed that, at P5, the circadian rhythms of clock genes expression in mouse heart were roughly in same phase as found in SCN. This suggested that the synchronization of the daily rhythms between the SCN and the heart in the mouse happened at P5.

## Discussion

In this study, we observed the postnatal ontogenesis of the molecular clockwork in mouse suprachiasmatic nucleus and heart. The data revealed that the postnatal circadian rhythms of clock genes developed gradually. In mouse SCN, all the studied clock genes expressed daily rhythms at P5, while at P3 in the heart. The present study suggested that circadian oscillators in SCN matured differently from those in peripheral tissues, such as heart. This observation is in coincidence with the recent study which showed that the clockwork in the mouse pars tuberalis matured earlier than in the SCN [21].

As early as at P1, all the studied clock genes were already expressed in SCN and heart. However, in SCN, only Cry1 and Rev-erbα mRNA expressed clear circadian rhythms. And in heart, there were no clear rhythms. At P3, Cry1, Rev-erbα and Bmal1 mRNA showed circadian rhythms in SCN and all the studied clock genes expressed obvious oscillators in heart. At P5, the four clock genes expressed clearly circadian rhythms in the both tissues. Interestingly, we discovered the phase of the clock gene expression changed during the ontogenic development and the SCN synchronized the heart at P5.

It was reported that the rat SCN was formed and the circadian rhythms of physiologic activities such as 2-deoxyglucose uptake and firing rate had present before birth [22], but clock genes in rat SCN showed on daily rhythms until at P3 [3]. The present study revealed that it was at P5 that all the studied clock genes expressed circadian rhythms. A plausible explanation of our data may be that the number of synapses increases slowly in the early postnatal periods and then dramatically between P4 and P10 [23], so the rhythms in clock gene expression were mutually synchronizing because of the adequate synapses. Light-induced endogenous oscillation is the most potent signal for synchronization of the clock genes in rodents and the pathway from the retina to the SCN is involved in the process of photic entrainment [8,24,25]. Previous reports had suggested that photoperiod is a major zeitgeber for entrainment of rodents' circadian rhythms [26]. Light induction of the FOS- like protein in the retina and SCN occurred first at P4 and the SCN can be stimulated by photic inputs as early as day 4 after birth[27]. Thus another explanation may be that the photoperiod by retina afferents acts as a major zeitgeber to affect the expression of clock genes at P5. The discrepancy between our data and previous report might be explained not only by a species difference between mouse and rat but also the experimental cues.

Our data indicated that the mRNA level of clock genes in heart expressed circadian rhythms at P3 and the rhythms more significant at P5. The finding suggested that autonomous circadian oscillation in the heart occurred as early as P3. On account of the absence of rhythms in all the studied clock genes expression, the synchronizing cue for peripheral clock oscillation might be independent of the SCN at the beginning of postnatal days. Food intake is an important zeitgeber for the circadian rhythms of peripheral clock genes [28]. The mouse mother nurses her infants periodically mainly in her rest time, during the day and she could feed herself during the night [29]. The restricted regimen of pups may synchronize the daily rhythms of peripheral clock. However, there existed another possibility that peripheral rhythms in the heart of infantile mouse were driven by the mother through directly and indirectly pathways. Circadian rhythms of pups were entrained by maternal cues during the first postnatal week [22,30]. Recent report had revealed that Per1, Bmal1 and Dbp mRNA in rat heart started circadian expression between P2 and P5 [20]. Thus the present study ascertain the exact day that circadian oscillator in mouse heart occurred as early as P3.

The difference of development of ontogenic clock genes expression between the SCN and the heart could be explained by tissue specificity: circadian oscillations mature at different rates in different tissues [31]. The clock genes of fetal monkey are differentially expressed in different tissues [32]. It was at P5 that rhythms of clock genes in SCN were roughly in the same phase with those in heart. Moreover, the phases of clock genes expression in the both tissues are similar to those in adult mouse. This observation may suggest that the SCN and the peripheral tissues such as heart oscillators start to be synchronizing as early as P5. It is possible that rhythmicity in the heart was driven by the SCN before P5. Nevertheless, because of the effects of insufficient synapses, hormone changes such as withdrawal of maternal melatonin and Avp, which are important for synchronization of slave oscillators in the brain and periphery of foetus [29], and maternal feeding habits at the early postnatal days, the entrainment of SCN to peripheral tissues was not so marked. With the retreating of maternal effects, the infantile mice gradually expressed their autonomous rhythms of clock genes. Furthermore, the phase changed as the maternal and autochthonous factors interact with each other during the ontogenic day. At P5, more stabile SCN neuronal network and initial innervation by retinal afferents might facilitate maturation of the SCN circadian rhythm generator[21]. Thus, as the master clock, the SCN can synchronize the peripheral clock more effectively.

In summary, we found that the circadian rhythms of the SCN and the heart in mice expressed respectively at P5 and P3, moreover, the SCN synchronized the peripheral clock at P5. The results might help us know better about the function of master and peripheral clock in animals. It is necessary to conduct a further investigation to reveal the profound mechanism.

## Materials and methods

### Animals

Female C57BL mice were housed in a temperature of 2 4 ± 2°C with free access to food and water. They were maintained separately on a 12L:12D light-dark cycle. According to Zeitgeber time (ZT), ZT0 is defined as lights-on time and ZT12 as lights-off time. Two white fluorescent tubes were used as source of light and illumination was between 100 and 200 lux depending on the position of cages. Day of delivery was designated the postnatal day 0 (P0). For postnatal studies of P1, P3 and P5, infants were kept with their mother through the experiment. Mothers with their pups were released into constant darkness 1 day before sacrificed. Then, three pups of mixed sexes were sampled at at different time points including ZT0, ZT4, ZT8, ZT12, ZT16 and ZT20. Hearts were harvested, frozen quickly in liquid nitrogen. Brains were taken away from the skull. And SCN was dissected grossly, quick-frozen in liquid nitrogen and stored at -80°C until RNA isolation. Animals were cared for according to the criteria of the Medical Laboratory Animal Administrative Committee of Shanghai.

### RNA isolation and real-time PCR

Total RNA was extracted with Trizol Reagent (Invitrogen, Carlsbad, CA). Total RNA was transcribed and amplified using ReverTra Ace qPCR RT Kit(TOYOBO, Osaka, Japan) according to the users manual, and real-time PCR was performed and analyzed using with SYBR-Green Real-time PCR Master Mix (Toyobo, Osaka, Japan). Each sample was measured in triplicate to ensure the accuracy of the datum. The relative expression level was determined with comparative CT method to normalize target gene mRNA to GAPDH mRNA. Primer sequences for PCR amplification are listed in Table 1.

**Table 1:** Primer pairs used to amplify PCR products.

Gene name	Annealing temperature	GenBank **accession no**.	Forward primer(5'-3')
mBmal1	60°C	NM_007489	Forward:CACTGACTACCAAGAAAGTATG Reverse: ATCCATCTGCTGCCCTGAGA

mCry1	58°C	NM_007771	Forward:CACTGGTTCCGAAAGGGACTC Reverse: CTGAAGCAAAAATCGCCACCT

mPer2	58°C	NM_011066	Forward:CAGACTCATGATGACAGAGG Reverse:GAGATGTACAGGATCTTCCC

Rev-erbα	61.2°C	NM_145434	Forward:AGTCGCTGACACTACACAGG Reverse:CCAGGTGGTGAAGGTATCTCC

GAPDH	55°C	BC_083149	Forward:ACAGCCGCATCTTCTTGTGCAGTG Reverse:GGCCTTGACTGTGCCGTTGAATTT

### Statistical analysis

The results are expressed as the means ± SEM and analyzed by one way ANOVA following Games-Howell post-test using SPSS 11.5 software. P < 0.05 was required to be statistically significant.

## List of abbreviations

SCN: suprachiasmatic nucleus; P: postnatal day; Bmal1: Brain and muscle ARNT-like protein 1; Per2: Period2; Cry1: Cryptochrome 1.

## Competing interests

The authors declare that they have no competing interests.

## Authors' contributions

JH carried out all aspects of experiments and data analysis, and drafted the manuscript. CL participated in the figure formatting and performed the statistical analysis. LH participated in the design of study and proofread manuscript. RQ conceived of the study and performed the experimental instruction.

All authors read and approved the final manuscript.
